# Perfusion MRI Indexes Variability in the Functional Brain Effects of Theta-Burst Transcranial Magnetic Stimulation

**DOI:** 10.1371/journal.pone.0101430

**Published:** 2014-07-03

**Authors:** Caterina Gratton, Taraz G. Lee, Emi M. Nomura, Mark D’Esposito

**Affiliations:** Helen Wills Neuroscience Institute and Department of Psychology, University of California, Berkeley, California, United States of America; Hangzhou Normal University, China

## Abstract

Transcranial Magnetic Stimulation (TMS) is an important tool for testing causal relationships in cognitive neuroscience research. However, the efficacy of TMS can be variable across individuals and difficult to measure. This variability is especially a challenge when TMS is applied to regions without well-characterized behavioral effects, such as in studies using TMS on multi-modal areas in intrinsic networks. Here, we examined whether perfusion fMRI recordings of Cerebral Blood Flow (CBF), a quantitative measure sensitive to slow functional changes, reliably index variability in the effects of stimulation. Twenty-seven participants each completed four combined TMS-fMRI sessions during which both resting state Blood Oxygen Level Dependent (BOLD) and perfusion Arterial Spin Labeling (ASL) scans were recorded. In each session after the first baseline day, continuous theta-burst TMS (TBS) was applied to one of three locations: left dorsolateral prefrontal cortex (L dlPFC), left anterior insula/frontal operculum (L aI/fO), or left primary somatosensory cortex (L S1). The two frontal targets are components of intrinsic networks and L S1 was used as an experimental control. CBF changes were measured both before and after TMS on each day from a series of interleaved resting state and perfusion scans. Although TBS led to weak selective increases under the coil in CBF measurements across the group, individual subjects showed wide variability in their responses. TBS-induced changes in rCBF were related to TBS-induced changes in functional connectivity of the relevant intrinsic networks measured during separate resting-state BOLD scans. This relationship was selective: CBF and functional connectivity of these networks were not related before TBS or after TBS to the experimental control region (S1). Furthermore, subject groups with different directions of CBF change after TBS showed distinct modulations in the functional interactions of targeted networks. These results suggest that CBF is a marker of individual differences in the effects of TBS.

## Introduction

Transcranial magnetic stimulation (TMS) is a valuable technique that allows experimenters to temporarily disrupt or enhance function in a targeted brain region in healthy individuals [1], [2]. The combination of TMS and functional Magnetic Resonance Imaging (fMRI) allows for powerful inferences to be made regarding the causal interactions among brain areas [3], [4]. This enables neuroscientists to move beyond the mere correlational arguments that can be made with measures of functional connectivity from fMRI data, potentially leading to substantive advancements in our understanding of the dynamic nature of brain networks.

However, past studies have found that the effect of TMS on brain function can be extremely variable across individuals [5], including the physiological changes occurring locally at the stimulation site (e.g., [6]–[12]). Even after employing individualized TMS parameters (e.g., motor thresholds, stimulation sites), the influence of TMS on underlying tissue can differ due to a number of other reasons [5]. Some of these factors are related to biophysical interactions between TMS and the target region, such as the individual variability in the distance between the skull surface and cortex, the precise folding pattern of the underlying cortex, and the orientation at which the TMS coil is held relative to the underlying cortex (reviewed by [13]). Similarly, the effect of TMS can depend on the previous state of the participant (e.g., interactions with ongoing fluctuations [8], previous physical activity [14], time of day [15], hormonal state [16]). More stable, intrinsic differences among participants can also explain some of the variability of TMS effects. For example, age [12], genetic makeup [17], and the excitability of distinct neuronal populations within an individual [7] have all been shown influence the effects of stimulation.

In both primary motor and sensory cortical regions, individual variability in the effect of TMS on underlying cortex can be estimated by measuring either muscle activity or calculating sensitivity thresholds after TMS [1]. However, in multi-modal association regions, where the application of TMS could significantly advance our understanding of large-scale networks and the complex functional roles of the underlying cortex, attempts to index TMS variability are complicated by the fact that there is typically no simple behavioral measure of the properties of a single region.

This issue is particularly evident in the case of studies that seek to identify repetitive TMS-induced changes in intrinsic large-scale networks measured during resting state fMRI, where no behavioral measurements are collected. This method is becoming increasingly popular because of the ease of data collection, the inter- and intra-subject reproducibility of resting state networks [18], [19], and the ability to measure a large number of clinically and behaviorally relevant networks in a short period of time [20]–[23]. However, despite the growing interest in defining the properties of intrinsic networks, the inherent variability in the effects of TMS on cortical function has posed a significant challenge in resting state studies. Changes in resting state connectivity caused by repetitive TMS (rTMS) have only been found in a small subset of regions remote from the stimulation site [24], [25] or in predicted locations with variable responses across individuals [26].

It would be useful, therefore, to develop a physiological metric that tracks individual variability in the local changes caused by TMS, even in multi-modal regions. FMRI can be used to measure both the Blood Oxygen Level Dependent (BOLD) signal and regional Cerebral Blood Flow (rCBF). The BOLD signal is the most widely used measure in fMRI, due to its high signal to noise ratio and temporal resolution relative to other MR measures. Unfortunately, the effects of TMS as measured by local BOLD activity have been quite mixed [27]. When TMS is applied online, concurrently with fMRI, a number of studies have failed to find changes in BOLD signal under the sites of stimulation [28]–[31]. Studies that have observed local effects have often done so only at high intensities of stimulation ([28]–[34], but see [35]); in cases of motor cortex stimulation these have evoked movements that might themselves have caused activity increases [28]–[31]. Similarly, fMRI studies during the performance of a task after TMS was applied offline have produced varied results, with some groups reporting no local BOLD signal changes [36]–[39] and others observing changes during particular tasks [40]–[42]. Moreover, given the arbitrary nature of BOLD measurement units, it remains unclear how to effectively interpret local BOLD signal changes caused by offline rTMS during a resting state, without the comparison between conditions that occurs in task-based modeling.

In contrast, rCBF measures of brain perfusion may hold several advantages over the BOLD signal, including a more quantitative read-out (with a unit of measurement that refers to a physical quantity, ml/g/min, unlike the arbitrary units of the BOLD signal), a stronger ability to detect slow neuronal changes, and better comparability across scan sessions [43]. These advantages make perfusion fMRI a strong candidate for measuring TMS-induced physiological changes, particularly during resting state fMRI. In the past, a number of studies have shown that CBF changes (usually, but not exclusively, increases) at the site of stimulation ([44]–[49]; see review by [27]). Here, our aim was to extend these findings to multimodal regions from intrinsic networks to determine if perfusion fMRI can be used to track individual differences in the local physiological effects of rTMS and link them to changes in functional network interactions.

In this study, we applied rTMS to multi-modal regions of intrinsic networks while measuring local rCBF to determine how individual variability in this measure was related to network-level functional BOLD activity. We used continuous theta burst TMS (cTBS), a rTMS protocol that produces long-lasting inhibition of underlying cortical activity [50], in order to target three different locations: left dorsolateral prefrontal cortex (*L-dlPFC*), left anterior-insula/frontal operculum (*L-aI/fO*), or left primary somatosensory cortex (*L-S1*). The two frontal regions represent multi-modal areas thought to be critically involved in two separable intrinsic networks related to cognitive control [51], [52]. Additionally, a previous analysis of the BOLD data collected here has shown that cTBS to these regions leads to widespread changes in functional connectivity in frontoparietal and cingulate cortices [53]. L-S1, a region not involved in these intrinsic networks, was used as an experimental control to monitor for non-specific effects of TBS and time.

Our analyses sought to examine how individual variability in rCBF under the coil was related to the variability in functional network properties (i.e., resting state functional connectivity measured during separate, interleaved, BOLD scans) of the targeted regions. We hypothesized that rCBF changes at the site of stimulation would measure the magnitude of local neural disruption and that variability in this disruption could be used to predict network-level changes in functional connectivity. Using three different stimulation sites with different connectivity profiles and different scalp-to-cortex distances allowed us to determine both whether TBS-induced changes were specific to the targeted region and how generalizable our metrics were to a variety of different brain sites.

## Materials and Methods

### Ethics Statement

All participants provided written informed consent before participating and were compensated monetarily for participation. The procedures for this study were approved by the Committee for the Protection of Human Subjects at the University of California, Berkeley.

### Participants

Twenty-seven healthy right-handed participants (11 female, 18–31, mean age = 22.7 years) completed four separate combined TMS and fMRI sessions.

## Experimental Timeline (see Figure 1)

The first session was used to acquire an anatomical image for each participant and to map subject-specific TBS sites and motor thresholds. The subsequent three sessions included a pre-TBS period, during which one BOLD and one ASL scan were collected. This was followed by a 40-second period of theta-burst TBS outside the scanner, after which participants re-entered the scanner and completed two blocks each of alternating BOLD and ASL scans. A different TBS site was targeted in each session (with order counterbalanced across participants): left dlPFC, left aI/fO, and left S1. During scanning, a fixation cross was presented to the screen and participants were instructed to remain awake with their eyes open and to allow their mind to wander without focusing on any particular topic. During all scans, the participant’s wakefulness was monitored using an MR-compatible eye-tracking camera. Each session took approximately one hour to complete, and separate sessions were always completed at least a day apart. In a few participants (N = 1 for aI/fO TBS, N = 1 for dlPFC TBS, and N = 2 for S1 TBS), the second perfusion scan was missing due to participants either falling asleep (N = 2) or sessions exceeding their time limit (N = 2).

**Figure 1:**
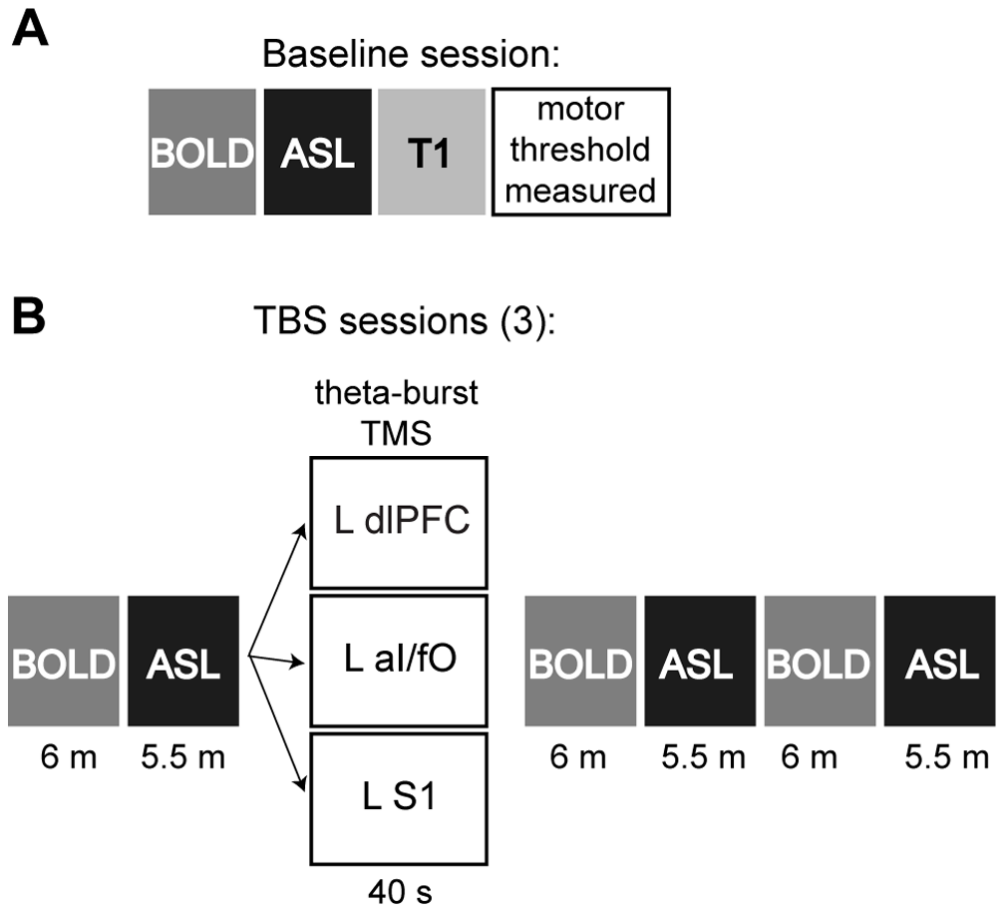
Experimental Timeline. Participants (N = 27) completed four separate scanning sessions. (**A**) The first session was used to acquire baseline BOLD and ASL fMRI measures, a high-resolution anatomical MRI scan, and resting motor thresholds using EMG. (**B**) The remaining three fMRI scans began with the collection of pre-TBS BOLD and ASL scans. These were followed by 40 s of theta-burst TMS to one of three target locations (L dlPFC, L aI/fO, or L S1). Following TBS, two BOLD and two ASL scans were collected in an interleaved fashion.

### Transcranial magnetic stimulation

All TMS stimulation occurred outside of the scanner. Subjects were seated in a comfortable chair throughout all TMS sessions. TMS was applied using a handheld figure-eight coil (outer winding diameter = 70 mm), and all pulses were delivered to the coil using a Magstim rapid stimulator with four booster modules producing biphasic pulses (*Magstim, UK*).

Motor thresholds were calculated during the first session. Electromyography was used to record potentials from electrodes placed on the first dorsal interosseous (FDI) muscle from the dominant hand. Stimulation was applied over the hand representation within primary motor cortex, with the coil placed tangentially to the scalp and the handle of the coil pointing posteriorly. This area was defined as the scalp location where stimulation led to the largest motor-evoked potential (MEP) from the contralateral FDI while the participant was relaxed. To define the stimulation intensity, the active motor threshold (AMT) was calculated in each participant. This was defined as the minimum intensity of single-pulse stimulation required to produce a detectable MEP from the contralateral FDI on 5 out of 10 trials while the participant maintained contraction at 20% of their maximum in the FDI. EMG signal feedback was provided through visual feedback on a computer monitor to help the participant maintain contraction consistently at this level. Motor thresholds were only taken once, to save time across the four days of combined TMS-fMRI measurements. Past studies have demonstrated that MEPs show low intra-individual variability across days, but high-inter-individual variability across subjects [54], suggesting that a single measurement per participant could reliably be used across the course of the experiment to produce robust measurements of TMS thresholds and equate stimulation levels across subjects.

Stimulation sites (defined as described below) were localized in each participant using a computerized frameless stereotaxic system (*Brainsight software, Rogue Research, Canada*). In this system, head position is defined in real time by using reflective markers that are placed on the participant’s head and imaged with an infrared camera. These positions are co-registered with anatomical locations placed on the individual participant’s structural MRI scan (acquired during the first session). Markers are placed both on the participant’s head and on the coil so that the relative position of the coil to the participant’s head (and MRI) can be tracked to precisely place the coil with respect to the targeted anatomical locations.

Once the coil was placed at the targeted location in each participant, 40 seconds of continuous theta-burst stimulation was applied at 80% of each individual’s AMT. During theta-burst TMS, 50 Hz trains of three TMS pulses are provided every 200 ms continuously over a period of 40 s (total of 600 pulses). This form of rTMS was selected to produce long-lasting reductions in cortical excitability that persist for up to 60 minutes following stimulation (as described by [50]) to allow for sufficient fMRI recording time, with the stimulation parameters used in this study matching those used by Huang and colleagues [50].

### Stimulation sites

**Figure 2:**
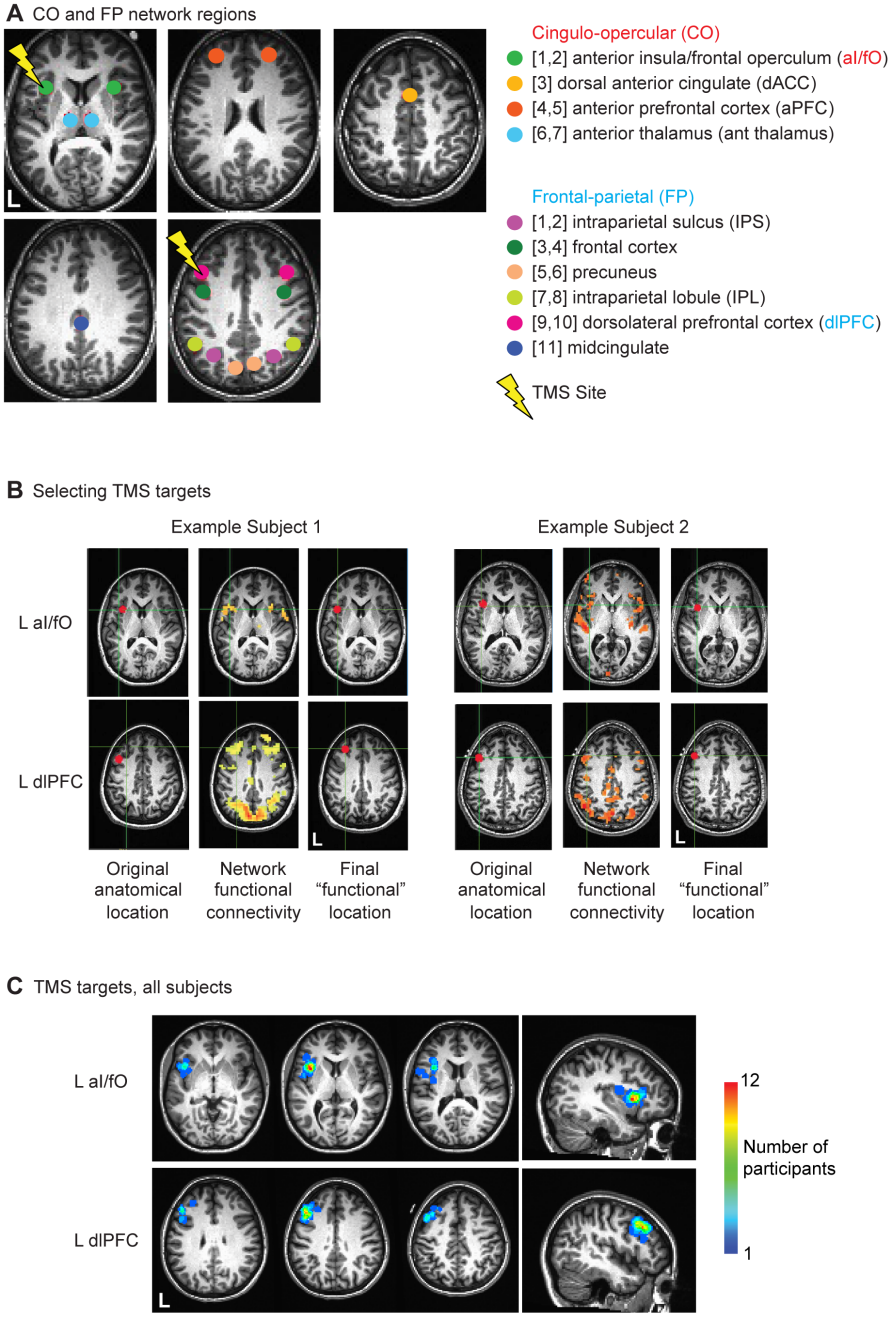
Regions of Interest. (**A**) Two of the TMS target regions were part of intrinsic cognitive control networks: the L dlPFC (in the fronto-parietal or FP network) and the L aI/fO (in the cingulo-opercular or CO network). The other nodes from these networks are also displayed. In addition, S1 was used as a control target region (*not shown*). (**B**) The L dlPFC and L aI/fO TMS targets were functionally localized in each subject by finding the cluster of within-network functional connectivity closest to previously published coordinates [52], *see Stimulation Sites*). In some subjects (e.g., *Example Subject 2*), these clusters were already near the predefined coordinates, but in others this process shifted target coordinates (e.g., *Example Subject 1*). (**C**) An overlap plot displays the relationship between L aI/fO (*top*) and L dlPFC (*bottom*) TMS targets across all individual participants on a normalized brain.

Three sites of interest were targeted in each individual (see ***Figure 2A***). Two sites were chosen from pre-defined intrinsic cognitive control networks [51], [52] to examine local and network-level effects of stimulation: the left dorsolateral prefrontal cortex (L dlPFC) and the left anterior insula/frontal operculum (L aI/fO). The third site, the left primary somatosensory cortex (L S1), was chosen to control for any changes due to nonspecific stimulation of brain tissue as well as the scalp sensation of stimulation. This region is not part of the two intrinsic cognitive control networks under study [52]. Stimulation sites were defined for each individual based on a combination of anatomical and functional criteria (modeled after [26], [55]) in an effort to provide individualized locations that best targeted the subject-specific locations of the intrinsic networks.

The control site (L S1) was established anatomically, by centering a spherical region-of-interest (ROI) over the left postcentral gyrus in each individual. Each participant’s left S1 was identified on the anatomical scan, and ROIs were drawn as spheres with a radius of 6 mm centered 10 mm away from the midline and 6 mm from the top edge of the brain.

The other two sites were based on coordinates from the fronto-parietal (L dlPFC) and cingulo-opercular (L aI/fO) cognitive control networks first described by Dosenbach and colleagues [51], [52] and modified for each individual participant through a functional connectivity analysis (see ***Figure 2B*** and the following description). First, we reverse-normalized all 18 regions in the Dosenbach cognitive control networks (11 fronto-parietal and 7 cingulo-opercular) into individual subject space. In each participant, a whole-brain functional connectivity analysis was then conducted using the resting state BOLD fMRI data from the baseline session. The whole-brain functional connectivity analysis was conducted for each network separately, using the average time-series from all regions in each network (excluding the target stimulation site). For example, a cingulo-opercular whole-brain functional connectivity map was created by averaging the time-series from all cingulo-opercular regions except the L aI/fO ROI and then computing Pearson’s correlation coefficients between this average time-series and that of every voxel of the brain. Similarly, a fronto-parietal whole-brain connectivity map was created using the average time-series from all fronto-parietal regions except the L dlPFC ROI (*see below for more details on data preprocessing and functional connectivity measures*).

Then, within each network map (cingulo-opercular for the L aI/fO node and fronto-parietal for the L dlPFC node) we found the cluster of connectivity closest to the originally specified coordinates for the target region (*see* ***Figure 2B*** for examples from two participants). To find the closest cluster, we thresholded the fisher-transformed correlation maps across a range of thresholds from z = 0.3 to 1.3 in intervals of 0.05 and clustered the maps created at each threshold. For each identified cluster, we took the coordinates from the center of mass of that cluster (or the peak voxel if the center of mass did not surpass threshold). We then found the cluster (across any threshold) nearest to the original anatomically defined ROI. We used these coordinates as our targets for TBS. In a small subset of individuals (4/27 subjects for L aI/fO and 2/27 subjects for L dlPFC), we found no cluster within 30 mm from the anatomical ROI (in which case, the original anatomical location was used for the center of the ROI). Notably all cluster targets were defined before the start of any TBS sessions for that participant. These subject-specific ROIs were defined in an effort to optimize the stimulation site for functionally impacting each cognitive control network in each individual. The relative overlap across subjects of these functionally defined ROIs can be seen on a normalized brain in **Figure 2C**.

### MRI acquisition parameters

All Magnetic Resonance Imaging (MRI) data were acquired from a Siemens MAGNETON Trio 3-Tesla scanner using a 12-channel head coil. Structural images were acquired during the first session using a whole-brain MP FLASH T1-weighted scan. Two forms of whole-brain functional MRI scans were acquired in each session: BOLD scans for measuring resting state functional connectivity and ASL scans for measuring resting state perfusion.

BOLD scans were obtained using a T2*-weighted EPI pulse sequence sensitive to blood oxygenation level-dependent (BOLD) contrast (TR = 2 s, TE = 24 ms, flip angle = 60 degrees, in-plane matrix = 64×64 pixels each 3.5×3.5 mm, with 37 3.5-mm descending axial slices and a 0.7 mm slice gap), and each scan consisted of 180 time points (6 minutes). Perfusion scans were collected using a pseudo-continuous ASL sequence with a standard EPI readout and PACE prospective motion correction [56], [57] (TR = 4 s, TE = 11 ms, flip angle = 90 degrees, in-plane matrix = 64×64 pixels each 3.4×3.4 mm with 20 5-mm descending axial slices and a 1 mm slice gap). Each scan consisted of 80 images (40 control/label pairs) and lasted about 5.3 minutes. Presaturation of fat was applied for both BOLD and ASL scans.

### Perfusion ASL fMRI Processing

#### Preprocessing

Preprocessing of the ASL scans was performed in SPM8 [http://www.fil.ion.ucl.ac.uk/spm/software/spm8/]. Data were extracted from DICOMs and then realigned to the first time-point of the entire session (day) using a rigid body (6 parameters) method.

#### CBF calculation

Cerebral blood flow (CBF) was calculated with software implementing a simple subtraction procedure and equation (1) from Wang et al [58], an equation based on the General Kinetic Model [59]. This generated CBF values for each time point. For mean CBF calculations, these values were averaged across all time-points within a scan.

#### Post CBF processing

After CBF calculation, the individual and mean CBF maps were coregistered to the anatomical image using the mean BOLD image produced from the ASL calculations to improve alignment (all alignments were manually checked; if coregistration with the anatomical image was poor, an individual motion-corrected ASL image was used as the target image). This step included resampling of data into 1 mm^3^ isotropic voxels. Data were then smoothed (6 mm FWHM) and gray-matter masked to exclude signals from high-intensity blood vessels outside of the brain. Finally, images were normalized by the global gray-matter CBF in each scan to produce regional CBF (rCBF) maps for the scan. Comparisons between pre- and post- TBS conditions from each day were taken with a simple difference.

### Resting state BOLD fMRI Processing

#### Preprocessing

Preprocessing for the resting state BOLD scans was carried out in AFNI [http://afni.nimh.nih.gov/afni/ [60]]. Images were extracted from DICOMs, and data were slice-time corrected (quantic Lagrange polynomial interpolation). Functional images were realigned to the first time-point in each session (using a rigid body 6-parameter method) and were coregistered to the anatomical image (using AFNI’s align_epi_anat.py).

#### Functional connectivity

Functional connectivity was computed using time-series correlations as in Fox et al [61]. Voxel time-series were bandpass filtered (0.009–0.08 Hz), in order to minimize physiological noise such as respiratory or cardiac artifacts, and smoothed (6 mm FWHM). In addition, we regressed out nuisance signals from subject-specific white matter and ventricle masks, each subject’s motion parameters, and their temporal derivatives. Functional connectivity was assessed by computing Pearson’s correlation coefficients between the average time-series from each ROI and every other voxel in the brain (for whole-brain analyses to determine TBS stimulation sites; *see Stimulation Sites section*) or between the ROI and all other ROIs in the same cognitive control network (in order to assess the functional properties of the ROI). These connectivity values were then Fisher-transformed before statistical analysis.

### Regions of Interest

The transformation from normal to native space was computed using SPM’s segment function [62]; this was then used to reverse-normalize relevant coordinates into native space. For the three sites targeted by TBS, regions of interest were determined as described under *Stimulation Sites*, based on a combination of anatomical and functional criteria for two cognitive control network ROIs [52] and a somatosensory cortex ROI. Additional ROIs used for functional connectivity analyses were defined using the reverse-normalized coordinates from Dosenbach [52].

### Statistical Analysis

Repeated-measures ANOVAs were carried out using SPSS (*Version 20, Chicago, IL*). First, an ANOVA with factors of time-point (pre-TBS or average of post-TBS blocks) and TBS condition (dlPFC, aI/fO, and S1) was performed in order to determine whether TBS consistently impacted rCBF measures under the coil. If a consistent change was found in a subset of TBS-conditions, this was further investigated with a repeated-measures ANOVA, with factors of time-point (pre-TBS vs post-TBS average), TBS condition, and ROI, in order to determine whether the TBS effects showed selectivity to the targeted TBS sites. Analyses initially compared the TBS effect averaged over the two post-TBS blocks and then investigated the duration of the effects by treating them separately for each block. In participants missing their second perfusion scan (see *Experimental Timeline*), the averages represented the values from their first post-TBS perfusion scan, and these participants were omitted from the block-specific analyses of the second block.

In addition, subsequent correlation analyses were conducted using Scipy [http://www.scipy.org/] to determine whether there was a relationship between the changes in rCBF measures and changes in functional connectivity of targeted nodes. Correlation values in these analyses represent Spearman’s (rho) instead of Pearson’s correlation coefficients, resulting in less influence from potential outliers. Differences between correlation values were evaluated using the formula described in Steiger [63] after using the conversion from Spearman’s to Pearson’s coefficients described in Myers & Sirois [64].

In accompanying analyses, subgroups of participants were selected based on the direction of perfusion change under the coil exhibited by those participants (increased or decreased perfusion). Changes in functional connectivity were visually plotted in unthresholded network plots. These were statistically compared by averaging the TBS-induced differences for all individual connections (fisher-transformed r-values) within a network and comparing between the two groups with paired t-tests. Similarly, the subset of connections linked to the TMS node were investigated by averaging all fisher-transformed r-values of connections linking the TMS node with the other nodes in it’s network and then compared across perfusion groups. All reported t-tests and correlations are accompanied by two-tailed p-values. Plots and network graphs were created using the NetworkX [http://networkx.github.io/] and Matplotlib [http://matplotlib.org/] packages in ipython [http://ipython.org/].

## Results

### Local changes in rCBF measurements under the TMS site

**Figure 3:**
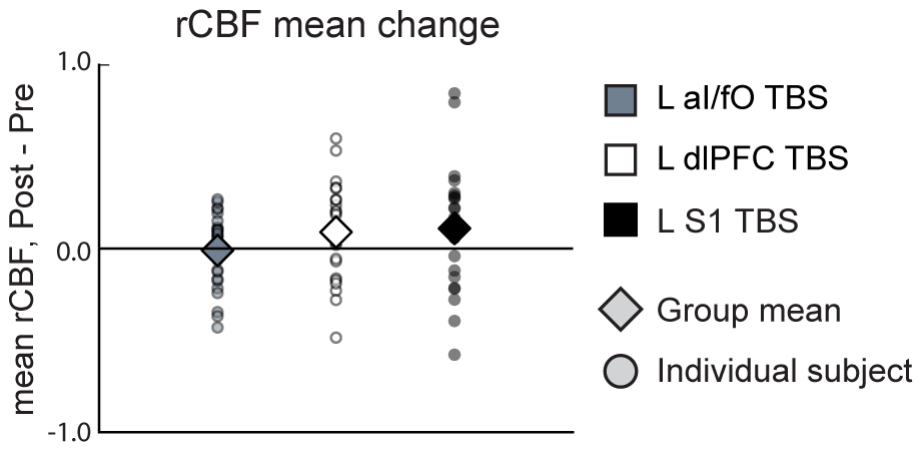
Individual variability in effects of TBS on rCBF measurements. Changes in mean rCBF under the coil are shown for each individual for each TBS conditions. As can be seen, individual variability was high, with subsets of participants showing increases and decreases in rCBF.

**Figure 4:**
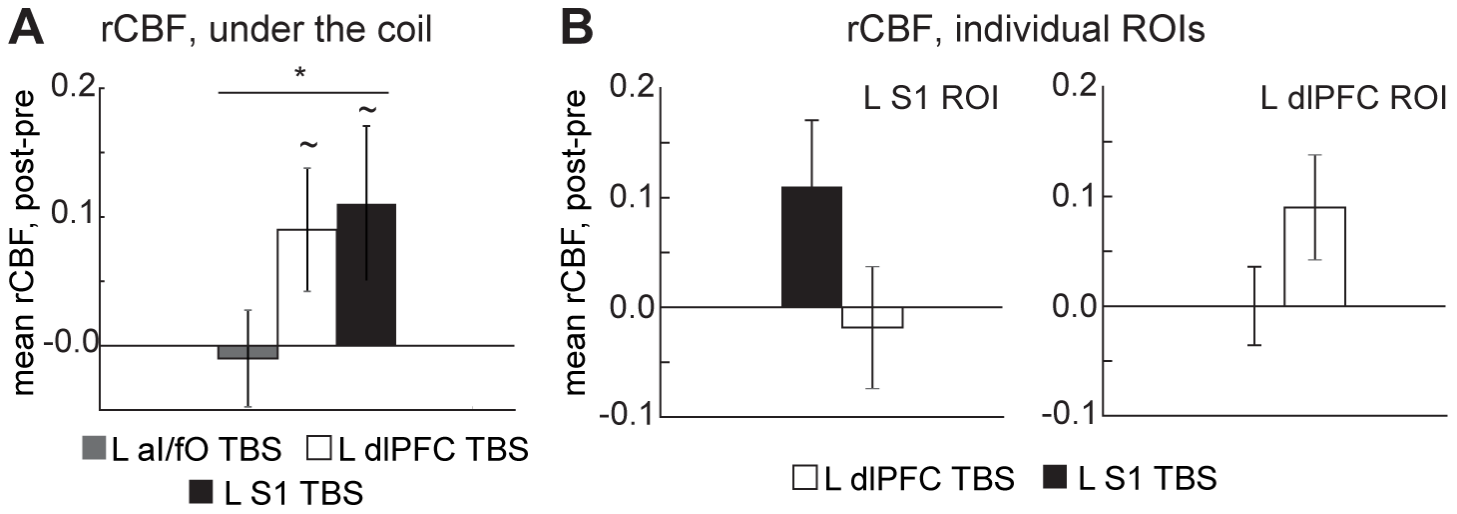
rCBF measures after TBS across all sites. (**A**) Changes in rCBF under the TMS site are shown for the three targeted locations. (**B**) At L S1 and L dlPFC, where consistent effects of TBS were seen under the coil, site selectivity was examined by comparing changes across the different targeted locations and regions of interest. (*p<0.05, ∼p<0.10; horizontal lines in ***A*** *indicate a significant main effect of block*).

As expected from previous work showing inconsistency in the local effects of cTBS across individuals, cTBS-induced changes in rCBF were weak on average across the group and variable in direction across participants at the targeted locations (aI/fO, dlPFC, and S1; see ***Figure 3***). Despite this variability, we observed a small, but significant increase in rCBF under the coil after cTBS relative to the pre-stimulation baseline across all stimulation sites (***Figure 4A***; *Main effect of block, F(1,26) = 4.61, p<0.05*]). At individual sites, a trend toward increases in rCBF under the coil were found after cTBS to left dlPFC [*t(26) = 1.88, p = 0.07*] and left S1 [*t(26) = 1.82, p = 0.08*]. These effects were slightly stronger during individual blocks [*dlPFC, Block 1: t(26) = 3.10, p<0.005; S1, Block 2: t(24) = 1.94, p = 0.06*] and the changes in mean rCBF under the coil for dlPFC and S1 were selective to the TBS site (*block x ROI x TBS-site interaction F(1,26) = 4.40, p<0.05;* ***Figure 4B*** *)*. However, the cTBS-induced changes seen under the coil for the left aI/fO region failed to reach significance [*average and both blocks individually: p>0.69*].

### Relationship between variability in local rCBF changes and changes in network activity after cTBS

Although there were some consistent cTBS effects on rCBF when averaged across subjects, individual participants showed variable direction and magnitude of changes in rCBF after TBS. Potentially contributing to this observed variability, the aI/fO region is deeper and more difficult to access with TMS than the other sites. This may have led to varying levels of stimulation both in this site compared to other sites and also across individuals whose individually selected sites (see *Methods*) were closer or further from the scalp. Therefore, we sought to determine whether variability in the local rCBF effects of cTBS is a functionally relevant measure of variability in the effects of stimulation on the underlying neural population or simply a consequence of imprecise/noisy rCBF measurements. One way to examine these individual differences in the efficacy of stimulation is by determining whether the cTBS-induced variability in these local measures is related to changes in the independent measurements of BOLD functional connectivity of our stimulated regions. Since the two frontal sites in our experiment were selected based on their roles within intrinsic cognitive control networks, we used these network interactions to probe the functional consequences of variability in cTBS effects.

#### Baseline correlations between rCBF and functional connectivity

**Figure 5:**
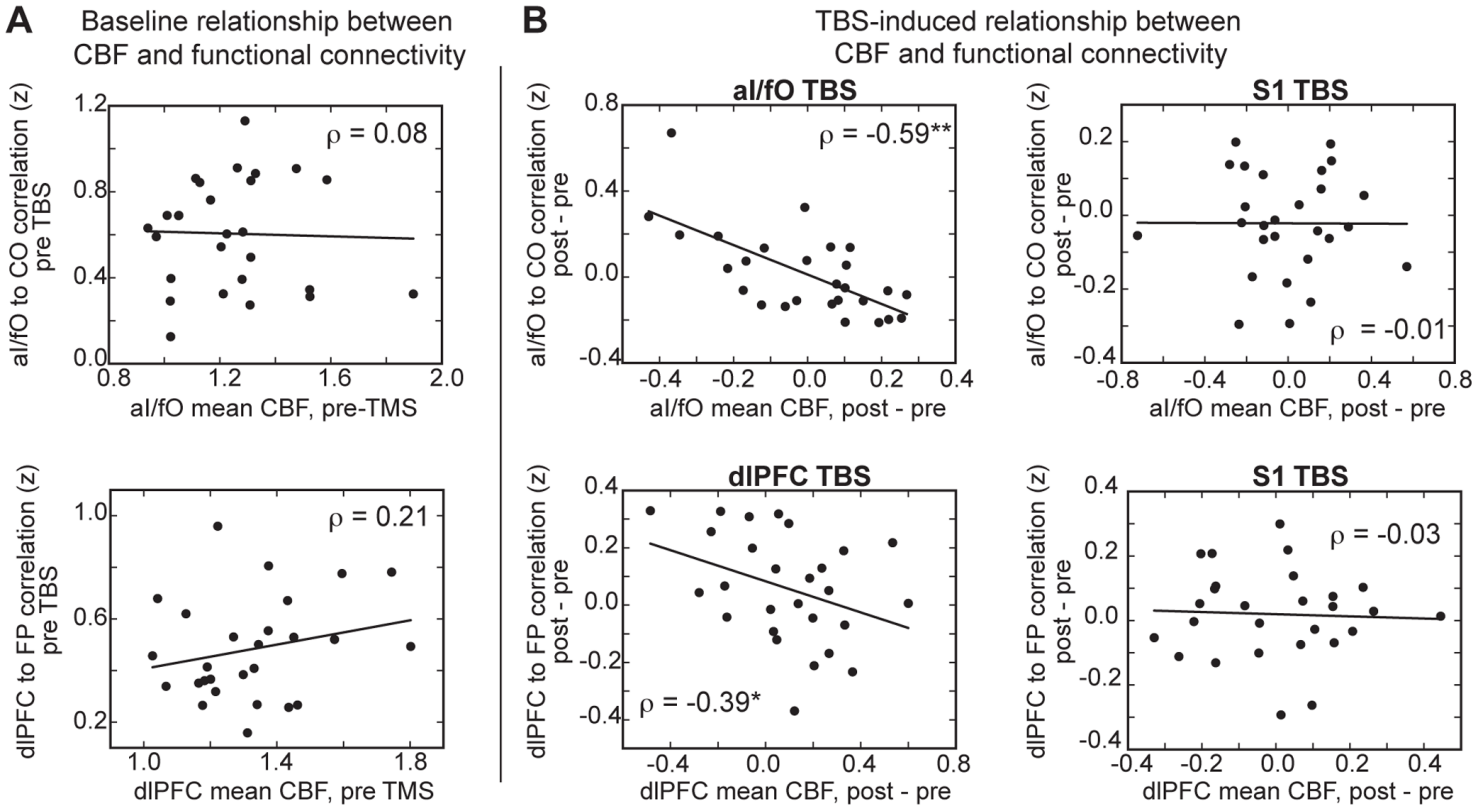
Relationship between rCBF and connectivity. (**A**) There was no relationship at baseline between rCBF and functional connectivity for either the aI/fO (*top*) or dlPFC (*bottom*) node. (**B**) Changes in aI/fO rCBF were related to changes in connectivity of aI/fO to the CO network after TBS to aI/fO (*top-left*) but not after TBS to S1 (*top-right*). Similarly, changes in dlPFC rCBF were related to changes in connectivity of dlPFC to the FP network after TBS to dlPFC (*bottom-left*) but not after TBS to S1 (*bottom-right*).

Regional CBF was not significantly related to the functional connectivity of each node prior to the application of cTBS. During the pre-TBS period, the rCBF of aI/fO was not related to the functional connectivity of the node to its own (cingulo-opercular) network [*aI/fO ROI: ρ = 0.08, p>0.69*]. Similarly, the rCBF of dlPFC was not related to the functional connectivity of the node to its own (fronto-parietal) network before TBS [*dlPFC ROI: ρ = 0.21, p>0.29*] (***Figure 5A***). This suggests that any discovered relationship between rCBF and functional connectivity following stimulation is due to changes in nodal and network-level activity caused by cTBS, not simply a consequence of an ongoing baseline relationship.

#### TBS-induced correlations between rCBF and functional connectivity

For both the aI/fO and dlPFC ROIs, the average change in rCBF after TBS was negatively related to the average change in functional connectivity between the stimulation site and its own network (*aI/fO to CO: ρ = *−*0.59, p<0.005;* dlPFC to FP: *ρ = *−*0.39, p<0.05*; see ***Figure 5B*** ***, left***). That is, increases in rCBF after TBS were related to decreases in the functional connectivity between the targeted region and its own network.

The relationship between changes in rCBF and BOLD functional connectivity was present in the aI/fO ROI only after TBS to aI/fO, not after TBS to S1 (the experimental control site) [*ρ = *−*0.01, p>0.97*], and these two relationships were significantly different from one another [*Z = *−*2.44, p<0.02*] (***Figure 5B*** ***, right***). Similarly, rCBF and functional connectivity for the dlPFC ROI were only related after TBS to dlPFC, not after TBS to S1 [*ρ = *−*0.03, p>0.87*]. Although the direct comparison between these correlations did not reach significance [*Z = *−*1.37, p = 0.171*], the relationships trended toward being significantly different from one another during the first block after TBS [S1 TBS: *ρ = 0.14, p>0.49*; S1 TBS vs dlPFC TBS: *Z = *−*1.92, p = 0.055*]. This suggests that variability in the local rCBF effect of TBS is selectively related to variability in the network-level functional properties of the stimulated region.

#### Participant subgroups selected based on perfusion

**Figure 6:**
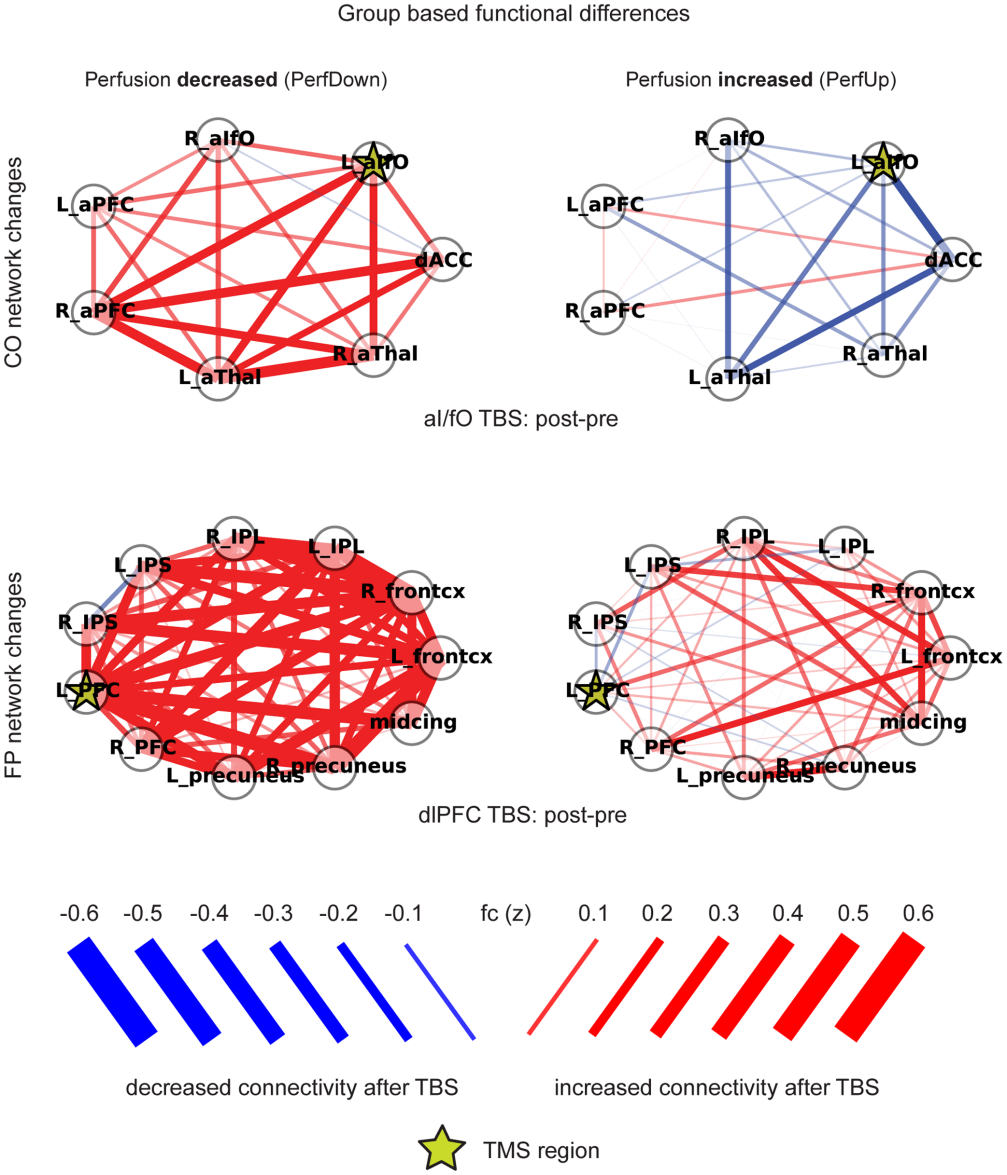
Connectivity in subject groups selected based on the direction of changes in rCBF following TBS. Participant groups were selected based on the direction of perfusion change exhibited by the participants: one group contained participants whose rCBF decreased under the site of TMS (*PerfDown*, *left column*), and the other contained participants whose rCBF increased under the site of TMS (*PerfUp*, *right column*). Unthresholded changes in functional connectivity within the CO network after aI/fO TBS (*top row*) and within the FP network after dlPFC TBS (*bottom row*) are displayed. Colors indicate the direction of change after TBS (*red = increases, blue = decreases*), and the width and transparency of the lines indicate the magnitude of changes (*wider/more opaque = higher magnitude changes*). Stars mark the TMS stimulation locations and the magnitude of functional connectivity changes is shown in the legend.

As expected given the correlation results above, when participants were selected based on whether perfusion increased (*PerfUp* subgroup) or decreased (*PerfDown* subgroup) under the TMS site, markedly different effects were seen in the connectivity of the TMS node with all of the other nodes in its functional network (***Figure 6***). After aI/fO TBS, the PerfDown subgroup trended toward increased connectivity between the aI/fO node and its associated CO network [*t(12) = 1.91, p = 0.08*] compared to the pre-TBS period. Instead, the PerfUp subgroup showed significantly decreased connectivity between that node and the CO network [*t(13) = *−*2.38, p<0.04*], and these subgroups showed significantly different changes in within-network functional connectivity from one another [*t(25) = *−*2.84, p<0.01*]. In addition, these subgroups showed significantly different changes across the entire CO network [*t(25) = *−*2.25, p<0.04*]. Similarly, after dlPFC TBS, the PerfDown showed significantly increased connectivity between L dlPFC and the associated FP network [*t(7) = 3.64, p<0.01*], as well as in general between all nodes across the entire network [*t(7) = 3.93, p<0.01*]. The changes in connectivity between dlPFC and its network (FP) were also significantly different between the PerfUp and PerfDown subgroups [*t(25) = *−*2.48, p<0.03*].

#### Relationship between local TBS-induced perfusion changes across sessions

**Figure 7:**
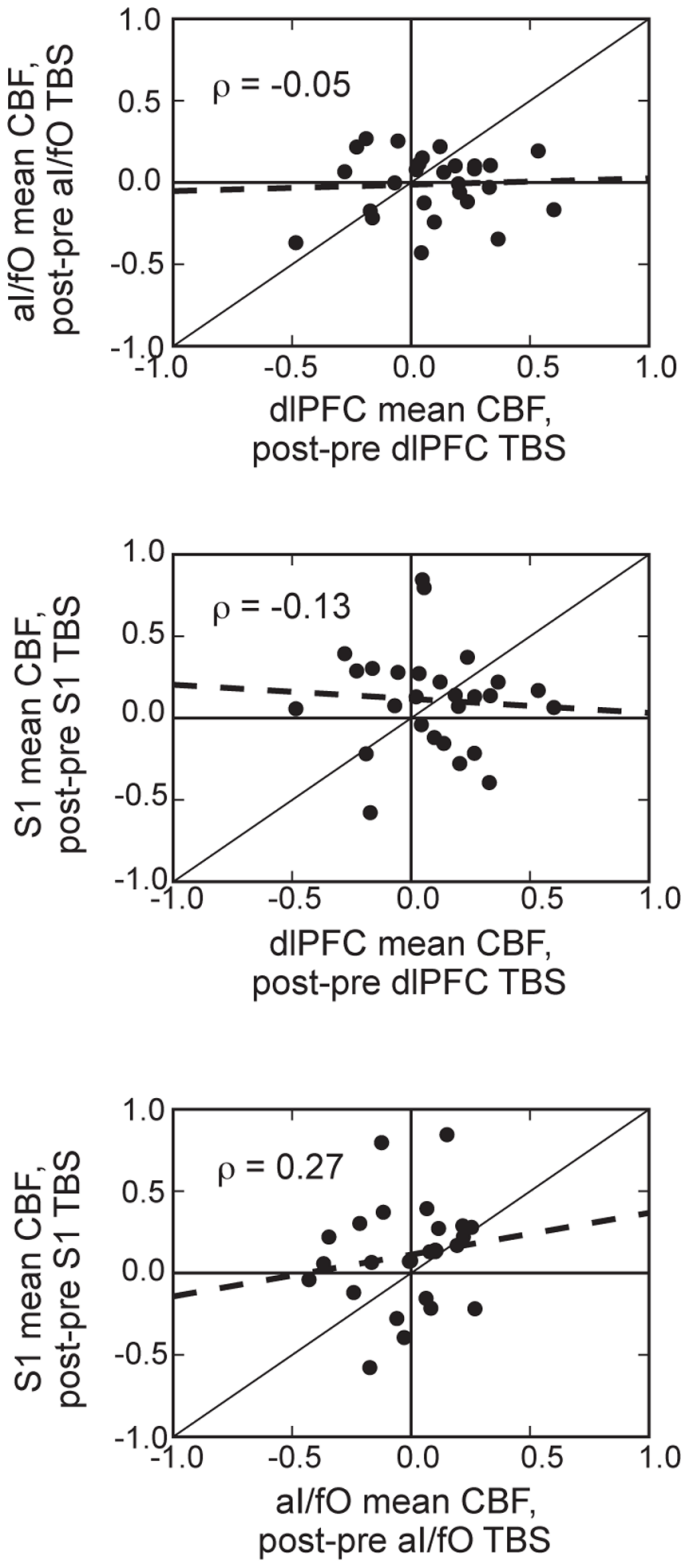
Relationship between TBS effects across sites. This figure shows the relationship between changes in rCBF under the coil for different TMS sites targeted on separate days, with each individual point representing a single participant. Changes in rCBF under the coil were not strongly correlated across different TBS locations (*dlPFC to aI/fO, top: r = *−*0.06; S1 to dlPFC, middle: r = *−*0.14; S1 to aI/fO, bottom: r = 0.26*). Dashed lines indicate the linear regression fit, and black diagonal lines indicate equality between the TBS effects on perfusion.

Many different factors may contribute to the variability in TMS effects. This variability may be primarily driven by stable differences in each individual subject’s susceptibility to TMS or by time-varying factors such as fluctuations in participants’ states, previous experiences, and the location of the targeted region (which differed across the three TMS session in our study). If certain participants are simply more susceptible to TBS than others, regardless of TBS site, then we might expect to see a relationship between the changes in perfusion under the coil across different sites. However, no such relationship was observed for any combination of stimulated locations (***Figure 7*** [*all |r_S_| <0.27, p>0.18*]). This suggests that global individual-based variables are not sufficient to explain the variability seen in the response to cTBS in this study.

## Discussion

The effects of rTMS can be quite variable across individuals and TMS sites [5], and it is often not clear how to directly measure this variability, especially when stimulation is applied over non-primary cortical regions during a resting state without corresponding behavioral measures. Here we examined changes in rCBF measures under the site of stimulation and whether these changes were linked to changes in functional connectivity of the stimulated region. The use of separate stimulation sites allowed us to examine the generality of these findings across TMS locations. We found that cTBS tends to show weak, selective, increases in rCBF under the site of stimulation. Significantly, variability in these increases was related to changes in the interactions of the targeted region with other nodes in its functional network. This relationship between perfusion and functional connectivity of the CO/FP networks was not present after cTBS to the region used as an experimental control, L-S1. Our findings show a link between variability in local alterations in brain function and long-range network interactions. This suggests that mean rCBF may be a useful independent variable for selecting participant groups that have differing functional effects of rTMS.

### Increased rCBF under the TMS site

Under the site of stimulation, rCBF slightly increased on average across individuals, particularly after L dlPFC and L S1 stimulation. Changes in rCBF after cTBS to the L aI/fO were more variable than cTBS to the other two regions, and this may be because this region is further from the coil and therefore more difficult to access with TMS. The increased rCBF after cTBS to L dlPFC and L S1 seems potentially counterintuitive, given the substantial physiological and behavioral evidence suggesting that the primary impact of continuous theta burst TMS is inhibitory [2], [65], [66]. Theta-burst TMS decreases the magnitude of evoked potentials in motor [50] and somatosensory [67] regions, decreases motor excitability [68], increases saccade latencies [69], and increases phosphene thresholds [70]. Furthermore, studies of theta-burst TMS have suggested that it alters inhibitory systems, as assessed with electrophysiological recordings from the spinal cord [68] and MR spectroscopy measurements [71] in humans, and with electrophysiological recordings and measurement of protein expression in rats [72].

However, past studies using inhibitory TMS protocols have also shown evidence for increased rCBF under the coil [Paired-pulse TMS: [73]; 1 Hz: [44], [45], [48], [74]; cTBS: [47]]. A number of potential explanations have been put forth for these findings. One possibility is that more neurons are recruited to maintain a constant level of performance after inhibitory TMS to a region [47], but this explanation may be difficult to interpret in this experiment given the lack of an explicit behavioral task. Others [45] have instead proposed that, while theta-burst TMS may drive a decrease in post-synaptic activity, excitatory pre-synaptic activity may be up-regulated in a compensatory fashion, leading to the observed increase in CBF. Finally, although it has been shown that intracortical inhibition can be decreased by TBS over motor cortex [50], it is also possible that the inhibition caused by theta-burst TMS may selectively increase the activity of metabolically demanding inhibitory neurons in multi-modal regions, which then increases CBF while concomitantly decreasing activity in excitatory neurons ([75]; as suggested by [45]).

### Local changes in CBF after TBS are related to changes in the functional network properties of the stimulated region

To determine whether the local variability we measured in rCBF was indexing functionally meaningful changes in the effects of TBS on the brain (rather than measurement variability), we examined whether there was a relationship between rCBF variability and changes in BOLD functional connectivity of the targeted region’s network. We found a specific association between these measurements that was evident only after cTBS to the experimental targets (and not a remote region, the control stimulation site, S1). Our findings raise the possibility that cTBS may affect individual subjects (and their underlying neural interactions) in qualitatively different fashions, perhaps along a functional continuum. rCBF measures showed both increases and decreases after cTBS in our study depending on the individual and stimulation site examined. This variability in the effect of stimulation on rCBF was coupled with the functional integration of the stimulated region in its own intrinsic network. Interestingly, this relationship was selective to changes induced by TBS; there was no relationship between rCBF and functional connectivity of the CO or FP networks after cTBS to a remote control location (S1), nor was there a baseline relationship before the application of TBS. Subgroups of participants selected based on the direction of local perfusion changes after TBS showed significantly different changes in network connectivity. This suggests that the relative balance between inhibitory and excitatory functional changes varies across individuals after stimulation, and that this may vary further depending on the exact target location and state of the participant. This variability may arise from differing contributions from the multiple proposed cellular and network mechanisms that are thought to accompany cTBS (described above).

Furthermore, the direction of the relationship between CBF effects and functional connectivity after TBS may initially appear somewhat counterintuitive, as we found that *increased* rCBF was related to relatively *decreased* functional connectivity of the stimulated region. As reported in our previous work using the same BOLD data analyzed here, functional connectivity tended to increase non-specifically across the networks associated with the stimulated regions after TBS [53]. However, here we show that perfusion increases coincided with relatively smaller increases in connectivity after TBS (or even slightly decreased connectivity, in the case of the CO network after aI/fO TBS). Decreases in perfusion, instead, were associated with relatively larger increases in functional connectivity. One possibility is that greater decreases in local activity (indexed by decreases in CBF) promote greater increases in compensatory network activity. In support of this idea, a recent study also found a similar inverse relationship between local and network measures after TMS: excitatory and inhibitory quadripulse TMS led to, respectively, decreased and increased functional connectivity of the stimulated region with contralateral areas. These effects were correlated, such that MEP reductions/increases were inversely proportional to the changes in functional connectivity [76]. Alternatively, if rCBF increases in this experiment are indexing local decreases in output activity (as discussed in the previous section; e.g., [45]), then increases in rCBF and relative decreases in functional connectivity may both be indicative of reduced neural processing. Note also that the direction of causality between the local rCBF effects and network connectivity cannot be inferred; rCBF may reflect the initial changes caused by TBS that lead in turn to connectivity changes or it may reflect feedback mechanisms from distal connected regions influencing the local TMS region. Future research utilizing invasive recordings may help to tease apart the mechanisms underlying the association between local rCBF and network connectivity.

In addition to our previous work [53], a handful of past studies have examined the effect of rTMS on resting state connectivity ([24]–[26], [77], [78], reviewed by [55]). Two of these studies only found relatively minor changes in the functional connectivity of a small set of regions remote to the stimulation site (in the form of both increased functional connectivity [25] and decreased connectivity [24]). Eldaief and colleagues found few consistent changes after inhibitory 1 Hz rTMS to the inferior parietal lobule (IPL, a component of the default mode network) and the only connectivity change surviving multiple comparisons was a significant increase in connectivity between IPL and the left hippocampal formation. However, decreased connectivity of IPL with the rest of its network was seen after 20 Hz rTMS, even though this type of TMS is thought to have a local excitatory action [26]. Note again that this fits with the results of the current work and other studies finding inverse relationships between the expected local effects of rTMS and changes in functional connectivity [76]. Additionally, we have previously reported that cTBS to aI/fO and dlPFC in this subject group led to increases in functional connectivity across widespread multi-modal regions in lateral frontoparietal and cingulate regions [53]. However, two recent studies add to the varied findings seen in resting state functional connectivity results when combined with rTMS: Chen and colleagues [77] found similar decreases in the functional connectivity of the default mode network after inhibitory 1 Hz stimulation of two different frontal regions (however, no changes were seen in the stimulated network and this remote decrease can not be distinguished from changes in functional connectivity that might occur naturally over time) and Rahnev and colleagues [78] found decreased connectivity during rest among visual regions after occipital compared with vertex cTBS. Here we add to this previous work by showing how local changes (measured with rCBF) are related to these network-wide effects. Furthermore, these findings suggest that rCBF may be useful in future resting state studies to select a subset of participants that respond similarly to TBS. However, although this method may help explain TBS-induced variability across participants, additional research will be needed to determine the mechanisms associating local perfusion changes with changes in functional connectivity and behavior before an unambiguous interpretation of differences between these subject groups can be made.

In our study, TBS-induced changes in functional connectivity and rCBF measurements under the coil were associated with each other, but this relationship was not evident at baseline, prior to TBS. One past study [79] has suggested that, at baseline, group-averaged functional connectivity and CBF values are positively related across regions, especially for long-range functional connections. Our study may not have had enough power to detect this baseline relationship (in addition, we did not examine the relationship across individual voxels as in [79]). However, in all cases examined in Liang et al. [79], the relationship between CBF magnitude and functional connectivity values was positive. This is not consistent with the relationship seen between TBS-induced changes in functional connectivity and perfusion measures in our study, which were negatively related. This may suggest that although local metabolic activity and long-range functional connections are generally in agreement with one another, this relationship may become fundamentally altered by external disruption. Past studies have found that local disruptions from focal brain lesions can also cause systematic changes in the functional interactions of resting state networks [80]–[83]. It is possible that other factors, such as task-based variables or internally driven goals, may differentially alter local metabolism and global functional coupling, selectively modulating this interaction at particular moments in time [79].

### Sources of individual variability in TMS effects

Following cTBS, rCBF measures were highly variable across participants in our study. This was true, even though stimulation thresholds and sites were individually optimized across participants, in an effort to increase the stability of our results (e.g., see [84]).

Variability in the effects of TMS have been reported across a wide variety of different stimulation protocols and locations [6]–[12]. Many factors have been proposed to contribute to this variability [5], and these can broadly be split into (1) static subject-specific variables (e.g., individuals’ age [12], genetic makeup [17], and variability in the activation of inhibitory interneurons of individuals [7]), and (2) fluctuating, site- or state-specific variables (e.g., previous physical activity [14], relationship between TMS and underlying electrophysiological fluctuations [8], [85], [86], geometry of underlying neural tissue [13], fluctuations in TMS thresholds across days and sites, and, in this study, variability in the time between TMS sessions). All of these factors could have contributed to the variability observed in our study. However, the lack of a significant relationship between the changes in rCBF under the coil across different sites suggests that subject-specific variables are unlikely to be a primary determinant of the variability in our study (***Figure 7***). This stands in contrast to a recent report that the ability to activate distinct neural populations accounts for a large amount of the variability in TBS effects in primary motor cortex [7]; however it is possible that these interactions between TMS and neuronal subpopulations may differ across target sites, in addition to individuals, and therefore may not be expected to correlate across stimulation days.

Site-specific influences on variability may have particularly contributed to TBS effects in the L aI/fO. Although this general region has been successfully targeted with TMS in the past [87] and produced reliable remote changes in functional connectivity in this subject group [53], this region is deeper and more difficult to stimulate than the other target areas. Variability in the exact position of this region, both in terms of depth and orientation of the cortical folding (and therefore in the orientations of cortical pyramidal neurons), may have added to variability in the local effects of cTBS. It is likely that the more superficial extents of the frontal operculum were the primary areas stimulated by TBS and that the extent to which disruption reached the peak region of this network node (which often lay deeper in the insula) differed across participants (e.g., ***Figure 2B and C***; note however that these more superficial regions are also components of the same large-scale cognitive control network, [*53*]). The coupling between changes in rCBF and in functional connectivity was particularly strong after TBS to the L aI/fO, supporting the idea that individual variability in the local and network effects of TBS may be predicted with rCBF measures.

### Conclusions

Together, our results suggest that rCBF is useful for measuring the local effects of TBS. In particular, we found that rCBF increases under the site of stimulation on average across participants. Furthermore, individual variability in rCBF is selectively related to changes in BOLD functional connectivity of the stimulated region. Finally, we found that variability in TBS effects was not correlated across experimental sessions on different days, suggesting that this variability is not primarily due to stable traits that differ across individuals.
